# Early virological response may predict treatment response in sofosbuvir-based combination therapy of chronic hepatitis c in a multi-center “real-life” cohort

**DOI:** 10.1186/s12876-015-0328-9

**Published:** 2015-08-04

**Authors:** Niels Steinebrunner, Martin F. Sprinzl, Tim Zimmermann, Marcus A. Wörns, Thomas Zimmerer, Peter R. Galle, Wolfgang Stremmel, Christoph Eisenbach, Kerstin Stein, Christoph Antoni, Jörn M. Schattenberg, Anita Pathil

**Affiliations:** 1 Department of Internal Medicine IV, University Hospital Heidelberg, Heidelberg, Germany; 2 Department of Internal Medicine I and Cirrhosis Center Mainz (CCM), University Medical Center Mainz, Mainz, Germany; 3 Department of Internal Medicine II, University Hospital Mannheim, Mannheim, Germany; 4 Department of Gastroenterology, Hepatology and Infectious Diseases, University Hospital of Magdeburg, Magdeburg, Germany

## Abstract

**Background:**

The combination of sofosbuvir (SOF), ribavirin (RBV) and peg-interferon-alfa-2a (peg-IFN-alfa-2a) as well as the combination of SOF and RBV for the treatment of patients infected with hepatitis c virus (HCV) has improved rates of sustained virological response (SVR) considerably in recent trials. However, there is only limited data concerning the efficacy and safety in a “real-life” cohort.

**Methods:**

We analyzed a cohort of 119 patients with chronic HCV infection treated at four investigational sites in Germany. All patients received either a combination treatment of SOF, RBV and peg-IFN-alfa-2a or SOF and RBV.

**Results:**

The rates of SVR at 12 weeks after end of treatment (SVR 12) were as follows: Among 76 patients with genotype 1 infection the SVR 12 rate was 74 % (*n* = 56), among 14 patients with genotype 2 infection the SVR 12 rate was 79 % (*n* = 11), among 24 patients with genotype 3 infection the SVR 12 rate was 92 % (*n* = 22) and among 5 patients with genotype 4 infection the SVR 12 rate was 80 % (*n* = 4). Of all 26 patients with a relapse in our cohort, 69 % (*n* = 18) of these patients presented with liver cirrhosis and 58 % (*n* = 15) were treatment experienced. Notably, the level of HCV-RNA after 4 weeks of treatment was a significant predictor of treatment response in genotype 1 patients. Patients with HCV-RNA levels ≥ 12 IU ml-1 after 4 weeks of treatment achieved SVR 12 only in 30 % (*n* = 17/56, *p* < 0.0001) of cases and treatment response was even lower with SVR 12 of 25 % (*n* = 5/20, *p* = 0.0016) in the subgroup of patients with cirrhosis.

**Conclusion:**

We observed a high rate of SVR 12 with SOF-based treatment regimes, however probably due to the high number of patients with liver cirrhosis and prior treatment experience, treatment response rates were lower than in previously published trials. In genotype 1 patients the analysis of early virological response may predict treatment response in SOF-based combination therapies.

## Background

Chronic hepatitis C virus (HCV) infection affects an estimated 170 million people worldwide with a prevalence of approximately 0.2–2 % in the United States and Europe [1, 2]. As HCV patients are at risk for developing end-stage liver disease with a variety of complications including hepatocellular carcinoma and decompensated liver cirrhosis with the need for liver transplantation, chronic HCV infection is associated with an elevated risk for liver-related mortality [3–5].

The next generation direct acting antiviral (DAA) sofosbuvir (SOF), which has been recently approved by national health authorities, represents the next milestone in the development of new therapeutic options and opens up potent treatment regimes for chronic HCV patients. SOF is an oral nucleotide analogue inhibitor of the HCV-specific NS5B polymerase with high antiviral efficacy and a favorable safety profile [6–8]. The efficacy of SOF-based treatment regimes has been demonstrated in different phase II and phase III trials [9, 10].

However, due to preselected patient populations and underrepresentation of difficult-to-treat patients, such as treatment experienced cirrhotics, these data may differ in a real-life setting and the validation of these results in a diverse patient population with less favorable conditions towards an SVR regarding concomitant diseases or constitutional factors may yield additional aspects and knowledge valuable for the future management of affected patients [11, 12].

Thus, we aimed to investigate the efficacy and safety of the SOF-based treatment regimes SOF, RBV and peg-IFN-alfa-2a or SOF and RBV alone in our “real-life” cohort from four tertiary referral centres in Germany.

## Patients and methods

### Patient population and study design

We analyzed clinical and laboratory data of all consecutive patients aged 18 years or older with treatment initiation for chronic HCV genotype 1, 2, 3 or 4 infection between January and June 2014 in a retrospective, longitudinal study at four investigational sites in Germany. One patient was non-adherent to the antiviral treatment plan and showed no SVR. This patient was included in the intention-to-treat (ITT) analysis.

Patients were treated with a combination treatment of SOF, RBV and peg-IFN-alfa-2a or SOF and RBV for either 12 or 24 weeks, depending on genotype, pretreatment history, presence of liver cirrhosis or contraindications according to the approved treatment recommendations [13]. SOF was administered at 400 mg once daily and RBV dose was based on body weight (1000 mg per day for <75 kg and 1200 mg per day for ≥75 kg in a divided dose) in all patients. Peg-IFN-alfa-2a was applied at a dosing of 180 μg once weekly to patients with genotype 1, 3 or 4 according to the individual treatment protocol. Serum HCV-RNA and standard laboratory tests were regularly assessed at baseline, at weeks 4, 12 and 24 of treatment and at additional time points, if deemed necessary, as well as at 12 weeks of follow-up. The lower limit of quantification (LLOQ) was 12 IU/ml (Abbott RealTime (ART) HCV assay (Abbott Molecular, Des Plaines, IL, USA). Liver cirrhosis was confirmed by liver histology or by evaluation of data sets from non-invasive tests, comprising fibroscan measurement, ultrasound examination, imaging by computed tomography or magnetic resonance, presence of esophageal varices and laboratory values. No patient with decompensated liver cirrhosis was included in the analysis. The institutional Ethics Committee (Ethikkommission der Medizinischen Fakultät Heidelberg) approved the protocol and the study was conducted in accordance with the Guidelines for Good Clinical Practice and the Declaration of Helsinki.

### Statistical analysis

Continuous data are expressed by mean values and standard deviation. Categorical variables are expressed as absolute and relative numbers. Continuous data over time was analyzed with one-sample *t*-test and categorical data with chi-square test. A p value <0.05 was considered statistically significant. Statistical analysis was performed using GraphPad Prism software (version 6.0, GraphPad Software, Inc., La Jolla, CA, USA).

## Results

### Characterization of the study population

We enrolled 119 patients with chronic HCV infection at four investigational sites in Germany. HCV genotype 1 was present in 64 % (*n* = 76) of patients, followed by genotype 3 in 20 % (*n* = 24), genotype 2 in 12 % (*n* = 14) and genotype 4 in 4 % (*n* = 5) of patients. The study population consisted of a large proportion of patients with liver cirrhosis 46 % (*n* = 55). Of all patients, 50 % (*n* = 60) were treatment experienced and 23 % (*n* = 27) had received a protease inhibitor in a previous therapy. The patient population comprised patients co-infected with human immunodeficiency virus (HIV) (*n* = 9) as well as patients after liver transplantation (*n* = 14). The distribution of patients after liver transplantation among genotypes was 7 patients with genotype 1 and 7 patients with genotype 3. Baseline characteristics of the study cohort are shown in Table 1. A combination treatment of SOF, RBV and peg-IFN-alfa-2a was administered to 68 % (*n* = 81) of patients and 32 % (*n* = 38) of patients were treated with SOF and RBV for 12 to 24 weeks. In detail, patients with genotype 1 received either a therapy regime of SOF + peg-IFN-alfa-2a + RBV over 12 weeks (43 % (*n* = 51)) or 24 weeks (10 % (*n* = 12)) or a therapy regime of SOF + RBV over 24 weeks (11 % (*n* = 13)). Patients with genotype 2 were exclusively treated with a 12-week regimen of SOF and RBV (12 % (*n* = 14)). A therapy regime of SOF + peg-IFN-alfa-2a + RBV over 12 weeks was applied in 12 % (*n* = 14) and of SOF + RBV over 24 weeks in 8 % (*n* = 10) of patients with genotype 3. In patients with genotype 4 the therapy regimes and treatment duration was SOF + peg-IFN-alfa-2a + RBV over 12 weeks in 3 % (*n* = 4) and SOF + RBV over 24 weeks in 1 % (*n* = 1) of cases (Table 2).

**Table 1:** Baseline characteristics of the study population

Demographics	
Mean age (years)	50 ± 12 (20–77)
Male sex	74 % (88)
HCV genotype	
1a	20 % (23)
1b	44 % (53)
2	12 % (14)
3	20 % (24)
4	4 % (5)
Mean HCV-RNA (10E6 IU ml-1)	3.23 ± 6.61 (0.02–34.50)
Cirrhosis	46 % (55)
Treatment history	
Treatment naive	50 % (59)
Treatment experienced	50 % (60)
Protease inhibitor experienced	23 % (27)
Clinical chemistry	
Platelets (10E3 μl-1)	155 ± 74 (13–396)
Total bilirubin (mg dl-1)	1.0 ± 0.8 (0.2–4.7)
INR	1.09 ± 0.25 (0.84–2.65)
Creatinine (mg dl-1)	0.78 ± 0.21 (0.43–1.85)

Data are expressed as percent (number) or means ± SD (range)

**Table 2:** Therapy regime and treatment duration

	GT 1	GT 2	GT 3	GT 4
SOF + PEG + RBV				
12 weeks	43 % (51)	0	12 % (14)	3 % (4)
24 weeks	10 % (12)	0	0	0
SOF + RBV				
12 weeks	0	12 % (14)	0	0
24 weeks	11 % (13)	0	8 % (10)	1 % (1)

Data are expressed as percent (number)

*GT* genotype, *SOF* sofosbuvir, *PEG* pegylated-interferon, *RBV* ribavirin

### Efficacy of sofosbuvir-based therapies

**Fig. 1:**
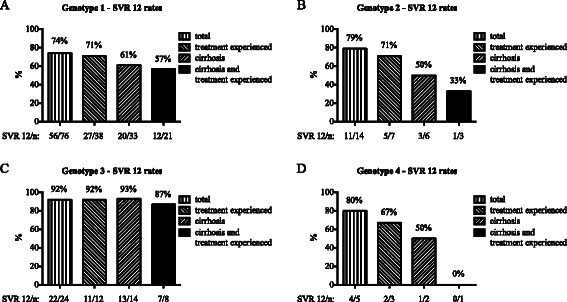
Efficacy of treatment of the study patients. Sustained virological response rates after 12 weeks after the end of treatment (SVR 12) are shown for patients with HCV genotype 1, 2, 3 or 4 (**a**–**d**). Patients were sub-classified for previous treatment experience or presence of cirrhosis. n = total number of patients

The SVR 12 rates according to the HCV genotype were as follows: Among 76 patients with genotype 1 infection the SVR 12 rate was 74 % (*n* = 56), 14 patients with genotype 2 infection had a SVR 12 rate of 79 % (*n* = 11), among 24 patients with genotype 3 infection the SVR 12 rate was 92 % (*n* = 22) and 5 patients with genotype 4 infection achieved a SVR 12 rate of 80 % (*n* = 4). Overall, 26 patients experienced a relapse in our cohort, 69 % (*n* = 18) of these patients presented with liver cirrhosis and 58 % (*n* = 15) were treatment experienced. The patient group with cirrhosis and previous treatment experience had the lowest SVR 12 rates in all four genotypes. Out of all 26 patients with a relapse in our cohort, 50 % (*n* = 13) presented with both negative predictors (Fig. 1).

In a subgroup of patients with genotype 1 HCV infection treated with SOF plus RBV and peg-IFN-alfa-2a for 12 weeks SVR 12 rates were 80 % (*n* = 41/51) and in those treated for 24 weeks SVR 12 rates were 75 % (*n* = 9/12). The results in both groups were not statistically different (*p* = 0.6779). However, it has to be considered that 11 out of 12 patients treated over a 24-week period with this regimen had cirrhosis and were treatment-experienced.

**Fig. 2:**
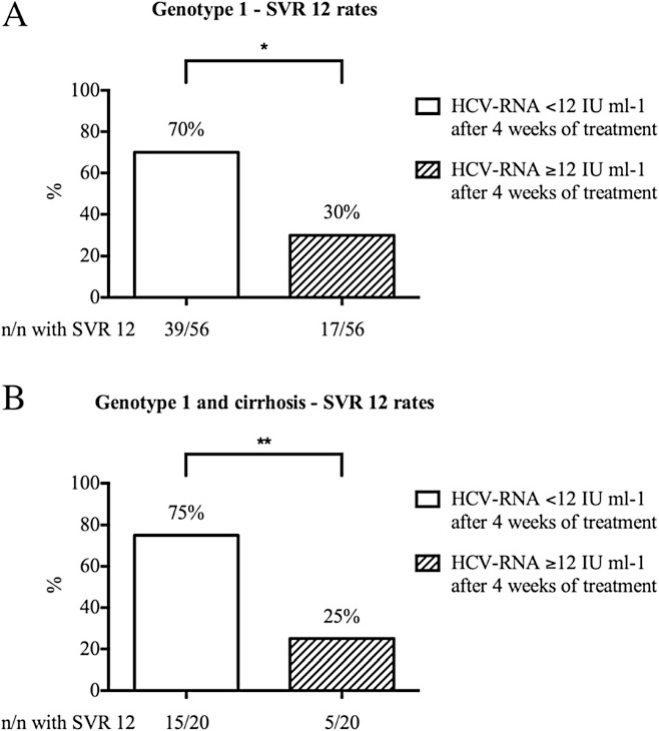
HCV-RNA level after 4 weeks of treatment as a predictor of response. Association of sustained virological response rates after 12 weeks after the end of treatment (SVR 12) of all patients with HCV genotype 1 infection with a level of HCV-RNA either < 12 IU –ml or ≥ 12 IU –ml after 4 weeks of treatment (**a**) and solely for patients with HCV genotype 1 and cirrhosis (**b**). n = number of patients. *p < 0.0001, **p = 0.0016

**Fig. 3:**
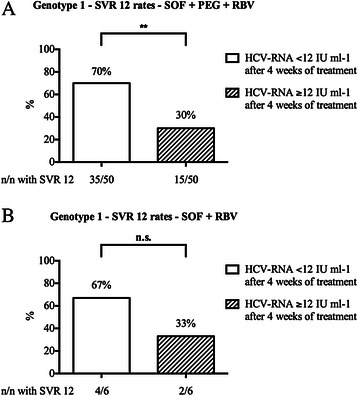
Association of RVR for SVR 12 with regard to either IFN-containing or IFN-free treatment regime. Association of sustained virological response rates after 12 weeks after the end of treatment (SVR 12) with RVR (level of HCV-RNA either < 12 IU –ml) for patients with HCV genotype 1 infection receiving either SOF + PEG + RBV (**a**) or SOF + RBV (**b**). n = number of patients. **p < 0.0001. n.s. = non significant

Further analysis indicated that besides the presence of cirrhosis, the level of HCV-RNA by week 4 of treatment was a significant predictor of treatment response in our genotype 1 population (Table 3). Patients with HCV-RNA levels ≥ 12 IU ml-1 after 4 weeks of treatment achieved SVR 12 only in 30 % of cases and treatment response was even lower with SVR 12 of 25 % in the subgroup of genotype 1 patients with cirrhosis (Fig. 2). Interestingly, HCV-RNA levels ≥ 12 IU ml-1 after 4 weeks of treatment was only associated with treatment failure in patients receiving an IFN-containing regime but not in patients on SOF + RBV (Fig. 3). In patients with HCV genotype 1 infection, there was a significant decline in total bilirubin levels when comparing the time points of treatment initiation and of SVR 12 (Table 4).

**Table 3:** Predictors of response for patients with HCV genotype 1 infection

	SVR 12	Relapse	p value
	total: 74 % (56)	total: 26 % (20)	
Age <65 years	95 % (53)	85 % (17)	0.1698
Age ≥65 years	5 % (3)	15 % (3)	
Female	34 % (19)	20 % (4)	0.2445
Male	66 % (37)	80 % (16)	
Non-cirrhotic	64 % (36)	35 % (7)	**0.0233**
Cirrhotic	36 % (20)	65 % (13)	
Treatment naive	52 % (29)	45 % (9)	0.6024
Treatment experienced	48 % (27)	55 % (11)	
HCV-RNA <6 (10E6 IU ml-1) at baseline	79 % (44)	85 % (17)	0.5352
HCV-RNA ≥6 (10E6 IU ml-1) at baseline	21 % (12)	15 % (3)	
HCV-RNA <12 (IU ml-1) after 4 weeks of treatment	70 % (39)	35 % (7)	**0.0065**
HCV-RNA ≥12 (IU ml-1) after 4 weeks of treatment	30 % (17)	65 % (13)	
Platelets <100 (10E3 μl-1) at baseline	18 % (10)	30 % (6)	0.2529
Platelets ≥100 (10E3 μl-1) at baseline	82 % (46)	70 % (14)	

Data are expressed as percent (number)

*SVR 12* sustained virological response at 12 weeks after end of treatment

Boldface data statistically significant

**Table 4:** Change from baseline to SVR 12 in patients with HCV genotype 1 infection

	Baseline	SVR 12	p value
Platelets (10E3 μl-1)	171 ± 81	173 ± 76	0.8913
Total bilirubin (mg dl-1)	1.2 ± 1.0	0.7 ± 0.4	**0.0052**
INR	1.03 ± 0.12	1.06 ± 0.21	0.4964
Creatinine (mg dl-1)	0.77 ± 0.18	0.80 ± 0.17	0.5529

Data are expressed as means ± SD

*SVR 12* sustained virological response at 12 weeks after end of treatment

Boldface data statistically significant

### Side effects of sofosbuvir-based therapies

In the treatment regimes consisting of SOF plus RBV and peg-IFN-alfa-2a as well as consisting of SOF plus RBV alone, the most common adverse events were fatigue and myalgia. No severe adverse events were reported. There was a significant difference in the reported side effect of hair loss in the two groups. With respect to hematologic abnormalities, anemia was most frequently observed, an event that is consistent with the well-known side effects of peg-IFN-alfa-2a and RBV. The rates of anemia, reduced white cell count and platelet count differed significantly between the two treatment groups. The SOF plus RBV therapy regime was generally well tolerated with fewer observed side effects as compared to the treatment with SOF plus RBV and peg-IFN-alfa-2a (Table 5).

**Table 5:** Adverse events and hematologic abnormalities

	SOF + PEG + RBV	SOF + RBV	p value
	total: 68 % (81)	total: 32 % (38)	
Adverse events			
Headache	5 % (4)	5 % (2)	0.9515
Fatigue	18 % (14)	16 % (6)	0.8170
Myalgia	15 % (12)	11 % (4)	0.5072
Hashimoto’s thyroiditis	5 % (4)	0	0.1608
Decreased appetite	3 % (2)	0	0.3256
Rash	5 % (4)	0	0.1608
Thrush	1 % (1)	0	0.4888
Hair loss	10 % (8)	0	**0.0435**
Aggressiveness	6 % (5)	0	0.1153
Pruritus	5 % (4)	0	0.1608
Insomnia	3 % (2)	3 % (1)	0.9662
Depression	3 % (2)	0	0.3256
Acute psychosis	1 % (1)	0	0.4888
Hematologic abnormalities			
Anemia (<10 g dl-1)	75 % (60)	37 % (14)	**<0.0001**
Leukocytopenia (<3 10E3 μl-1)	75 % (60)	16 % (6)	**<0.0001**
Thrombocytopenia (<100 10E3 μl-1)	60 % (48)	5 % (2)	**<0.0001**

Data are expressed as percent (number)

*SOF* sofosbuvir, *PEG* pegylated-interferon, *RBV* ribavirin

Boldface data statistically significant

## Discussion

The approval of SOF, the novel nucleotide analogue NS5B polymerase inhibitor, represents a breakthrough in the treatment of chronic HCV and has become the backbone of current therapy regimes. SOF-based therapies are the novel standard of care with high antiviral activity, broad genotypic coverage and a high barrier to resistance [6–8, 14, 15]. During therapy with SOF, no virological breakthrough has been reported so far [14, 16].

However, many difficult-to-treat patient populations hitherto have been understudied. Thus, we included a high number of patients with cirrhosis in our study, since HCV treatment represents a high priority particularly in this patient group. As HCV recurrence after liver transplantation is universal and bears a high risk of premature graft failure, we also analyzed patients after liver transplantation in our study. Previously, in the abovementioned patient groups, IFN-based HCV therapies were limited because of toxicity and poor efficacy [4]. Additionally, many patients in our study were treatment experienced and several of those had received a protease inhibitor in a previous therapy. The study population further comprised patients co-infected with human immunodeficiency virus (HIV). Recent data has shown that the outcome of DAA-based therapies in HCV/HIV co-infected patients is comparable to the HCV cure rates in HCV mono-infected patients and indication and drug choice should follow the general guidelines for HCV mono-infected subjects [17]. Therefore, HCV/HIV co-infected individuals are no longer regarded as a special patient population by major guidelines [13]. Instead, with current DAA-based therapies, genotype 3 infected patients or special populations, including patients with renal insufficiency or decompensated cirrhosis, have shifted into the focus as difficult-to-treat populations.

Considering overall SVR 12 rates, patients with HCV genotype 1 infection, which historically have been difficult to treat, still seem to be the population hardest to cure, as also reflected by our study results [10, 14, 17]. In the NEUTRINO trial, a phase 3 study in previously untreated patients with HCV genotype 1, a 12-week regimen of SOF plus RBV and peg-IFN-alfa-2a was administered. Total SVR 12 rates were 90 % and SVR 12 rates for patients with cirrhosis were 80 %. [14]. In a further small study involving treatment-naive patients with HCV genotype 1 infection and a high prevalence of advanced fibrosis and cirrhosis, a 24-week regimen of SOF and RBV resulted in SVR rates of 68 % [8, 18]. In our study population total SVR 12 rates were 74 % and SVR 12 rates for patients with cirrhosis were 57 %. While overall virological response rates are encouraging, the relative high relapse rate in genotype 1 patients may suggest that dual DAA combinations should be favored at least for patients with negative predictors for a successful treatment outcome.

Surprisingly, total SVR 12 rates for patients with genotype 2 were lower than expected. In contrast to the results in our study population, the combination of SOF plus RBV has yielded very favorable results in previous studies [9, 14, 19, 20]. In the FISSION trial, a phase 3 study involving previously untreated patients with HCV genotype 2 infection, a 12-week regimen of SOF and RBV showed total SVR 12 rates of 95 % and SVR 12 rates of 83 % for patients with cirrhosis [14]. In comparison, total SVR 12 rates for patients with genotype 2 in our collective was 79 % and was only 50 % regarding patients with cirrhosis.

Furthermore, in genotype 3 infected HCV patients, which have previously emerged as a particularly difficult to treat patient group, total SVR 12 rates in our study turned out to be higher than projected. The VALENCE trial yielded for previously treated and untreated patients with HCV genotype 3 infection total SVR 12 rates of 77–93 % after a 24-week regime of SOF and RBV, while the subgroup of previously treated cirrhotic patients displayed only SVR 12 rates of 61 % [9, 14, 16, 19]. With the addition of peg-IFN-alfa-2a to 12 weeks of SOF plus RBV in the LONESTAR-2 trial SVR 12 rates of 83 % were achieved in this unfavorable subgroup of previously treated cirrhotics [20]. It can be speculated that these positive results may reflect a selection of patients with early stage cirrhosis with only minimally lowered thrombocyte counts, which were therefore regarded to be eligible to receive IFN. The patients in our collective showed total SVR 12 rates of 92 % and even treatment-experienced cirrhotics showed a SVR 12 rate of 87 % despite an interferon-free treatment regime in most of the cases. Eventually these data suggest that besides SOF plus RBV for 24 weeks, a SOF plus RBV and IFN treatment for 12 weeks should still be considered for IFN-eligible genotype 3 patients.

The NEUTRINO trial, a study of SOF plus RBV and peg-IFN-alfa-2a in previously untreated patients with HCV genotype 4, presented total SVR 12 rates of 97 % and for patients with cirrhosis of 50 % [14]. Corresponding rates in our study population were 80 and 50 %.

However, it has to be noted that a comparison of the data of the aforementioned trials with the results of our study population is limited due to differences regarding the inclusion of treatment experienced patients and the treatment of patients with either one of two available treatment options and variable treatment duration.

Interestingly, besides the presence of cirrhosis, we observed that a level of HCV-RNA ≥ 12 IU ml-1 by week 4 of treatment was a predictor for treatment failure in genotype 1 patients, despite the fact that early virological response appeared to be of limited value as a prognostic marker in previously published DAA-based studies [14, 20]. Regarding the subgroup of cirrhotics, SVR 12 rates were only 25 % when HCV-RNA levels were ≥ 12 IU ml-1 after 4 weeks of treatment. Notably, the predictive value of early virological response was only evident in genotype 1 patients receiving an IFN-containing regime, but not in patients on SOF + RBV. Taken into account that SOF + peg-IFN-alfa-2a + RBV may still remain the standard of care in many regions of the world, because of the high costs of IFN-free treatment regimes, analysis of early virological response may be helpful to establish response-guided therapy regimes in the future. However, the sensitivity of HCV RNA quantification can differ between different tests. Patients who may have tested HCV RNA negative during antiviral therapy by older assays with a LLOQ of ≥ 50 IU/ml may test HCV RNA positive by highly sensitive HCV RNA assays. In our study the highly sensitive Abbott RealTime (ART) HCV assay (Abbott Molecular, Des Plaines, IL, USA) with the LLOQ of 12 IU/ml was used. Therefore, it might be reasonably assumed that different assays may have an influence on the predictive value of the early treatment response [21, 22].

Change in clinical chemistry from baseline to SVR 12 in patients with genotype 1 HCV infection showed a statistically significant decline in total bilirubin. It might be speculated that the observed change in total bilirubin under successful HCV therapy in genotype 1 patients could be translated into an improvement of the MELD score of patients with advanced stages of liver cirrhosis and therefore treatment may lead to a delay or permanent prevention of liver transplantation.

As a limitation of the study, there is no data on the interleukin 28B haplotype of the patients in our cohort available. An association of viral clearance with this polymorphism was shown for antiviral treatment with peg-IFN-alfa and RBV, but not for SOF so far [23, 24].

In our patient cohort no data is available on baseline and post-treatment resistance–associated variants (RAVs) representing a further limitation of our study. However, SOF exhibits a high barrier to resistance and among patients who did not achieve SVR in recent trials, including the FISSION, POSITRON and VALENCE trials, SOF resistance–associated variants (RAVs) were not detected [9, 14, 19]. On the other hand, the Q80K variant conferring resistance to the NS3 protease inhibitor simeprevir has been observed in 9-48 % of untreated HCV genotype 1a-infected patients, leading to reduced SVR rates [25]. Although patients with baseline RAVs still exhibit high SVR rates, screening for variants conferring resistance may help to reduce treatment failures with respect to cost intensive treatment regimes.

Consistent with the safety profile of IFN, adverse events and laboratory abnormalities were more common in the SOF, RBV and peg-IFN-alfa-2a group. As seen in our study, adverse event profiles improve substantially in the absence of IFN. In view of the inconvenience and high rate of significant side effects of IFN, all-oral, IFN-free DAA therapies will become the first choice for treatment of patients with chronic HCV.

However, even though great strides have been made since the approval of the first DAAs in 2011, future research needs to address the current limitations in the antiviral efficacy of available therapies in the increasing number of patients with advanced liver disease and previous DAA treatment failure. With a growing number of patients who have failed under DAA-based therapy, there is still an emerging demand for further novel antiviral agents.

## Conclusion

SOF-based therapy regimes are safe and lead to high rates of SVR 12. However, probably due to a large proportion of patients in our cohort with unfavorable conditions such as liver cirrhosis or pre-treatment failure or even the combination of both, the SVR rates of previous clinical trials were not attained in our patient cohort.

## Abbreviations

DAA: Direct-acting antiviral
EASL: European association for the study of the liver
HCV: Hepatitis c virus
HIV: Human immunodeficiency virus
ITT: Intention-to-treat
LLOQ: Lower limit of quantification
PEG: Pegylated-interferon
peg-IFN-alfa-2a: Pegylated-interferon-alfa-2a
RAV: Resistance–associated variant
RBV: Ribavirin
RVR: Rapid virological response
SD: Standard deviation
SOF: Sofosbuvir
SVR: Sustained virological response
